# The Possible Pathophysiological Outcomes and Mechanisms of Tourniquet-Induced Ischemia-Reperfusion Injury during Total Knee Arthroplasty

**DOI:** 10.1155/2018/8087598

**Published:** 2018-11-05

**Authors:** Prangmalee Leurcharusmee, Passakorn Sawaddiruk, Yodying Punjasawadwong, Nipon Chattipakorn, Siriporn C. Chattipakorn

**Affiliations:** ^1^Neurophysiology Unit, Cardiac Electrophysiology Research and Training Center, Faculty of Medicine, Chiang Mai University, Chiang Mai, Thailand; ^2^Department of Anesthesiology, Faculty of Medicine, Chiang Mai University, Chiang Mai, Thailand; ^3^Center of Excellence in Cardiac Electrophysiology Research, Chiang Mai University, Chiang Mai, Thailand; ^4^Department of Oral Biology and Diagnostic Sciences, Faculty of Dentistry, Chiang Mai University, Chiang Mai, Thailand

## Abstract

Ischemia and reperfusion (I/R) injury induced by tourniquet (TQ) application leads to the release of both oxygen free radicals and inflammatory cytokines. The skeletal muscle I/R may contribute to local skeletal muscle and remote organ damage affecting outcomes after total knee arthroplasty (TKA). The aim of the study is to summarize the current findings associated with I/R injury following TKA using a thigh TQ, which include cellular alterations and protective therapeutic interventions. The PubMed database was searched using the keywords “ischemia reperfusion injury,” “oxidative stress,” “tourniquet,” and “knee arthroplasty.” The search was limited to research articles published in the English language. Twenty-eight clinical studies were included in this qualitative review. Skeletal muscle I/R reduces protein synthesis, increases protein degradation, and upregulates genes in cell stress pathways. The I/R of the lower extremity elevates local and systemic oxidative stress as well as inflammatory reactions and impairs renal function. Propofol reduces oxidative injury in this I/R model. Ischemic preconditioning (IPC) and vitamin C may prevent oxygen free radical production. However, a high dose of *N*-acetylcysteine possibly induces kidney injury. In summary, TQ-related I/R during TKA leads to muscle protein metabolism alteration, endothelial dysfunction, oxidative stress, inflammatory response, and renal function disturbance. Propofol, IPC, and vitamin C show protective effects on oxidative and inflammatory markers. However, a relationship between biochemical parameters and postoperative clinical outcomes has not been validated.

## 1. Introduction

Total knee arthroplasty (TKA) is a surgical treatment aiming at improving the mobility and quality of life of patients suffering from advanced knee osteoarthritis. The prevalence of this procedure has substantially increased in the past decade and is expected to continue [1, 2]. A hallmark of the clinical success of TKA is postoperative quadriceps muscle function. Muscle atrophy following a use of intraoperative thigh tourniquet (TQ) results in early postoperative deficits in quadriceps strength and subsequently impaired TKA rehabilitation. The majority of TKA patients are in the elderly population [1] whose TQ-induced muscle loss is likely permanent and may increase risk for falls as well as loss of independence [3].

A TQ is routinely used in extremity surgery to produce a bloodless surgical field. However, TQ application alters normal physiology and is associated with several complications [4]. Locally, a circumferential inflatable cuff compresses the structures beneath the cuff and can possibly cause mechanical and ischemic injuries to localized muscles and nerves. Skeletal muscles distal to the TQ are also affected at a molecular level by prolonged inadequate blood flow and subsequent restoration of circulation. Systemically, limb exsanguination followed by TQ inflation transiently increases central blood volume and systemic vascular resistance, induces a hypercoagulable state, and activates fibrinolytic activity. Clinically, the use of a TQ is considered a risk factor for thromboembolism [5]. However, the incidence of deep vein thrombosis and pulmonary thromboembolism after TKA was found to be similar regardless of the use of the TQ [6].

TQ inflation induces ischemia to an extremity, and its release may lead to an ischemia and reperfusion (I/R) injury to not only localized skeletal muscle but also systemic circulation and vital distant organs including the brain, heart, lungs, and kidneys. The restoration of blood flow following an ischemic period is essential to preventing irreversible cellular injury; however, the reperfusion can augment secondary damage to ischemia. During oxygen deprivation, intracellular ionic and metabolic changes including ATP depletion, intracellular acidosis, and cytosolic calcium overload occur and cause damage to cells [7]. In addition, ischemia can exacerbate reactive oxygen species (ROS) production and promote a proinflammatory state, which subsequently increases tissue vulnerability to further injury during reperfusion. Upon the reintroduction of oxygen, excessive production of ROS disproportionate to the antioxidant capacity results in cell injury through the oxidation of proteins, lipids, and DNA.

Several treatment strategies have been proposed to prevent or attenuate the effects of I/R injury following TQ use in cases of orthopedic surgery [8]. Studies into the use of ischemic preconditioning (IPC) and antioxidants have generated inconclusive results depending on the administration techniques. Of the anesthetic agents, propofol is the best medication producing both antioxidative and anti-inflammatory effects. However, the correlation between the benefits of these interventional and pharmacologic strategies with the postoperative clinical outcomes has not been drawn.

Therefore, the aim of this review is to summarize current findings relating to the effects of TQ-induced I/R injury on localized skeletal muscles, local and systemic circulation, and remote organs in TKA surgery and therapeutic interventions in clinical study. Furthermore, the controversial reports regarding these issues are included and discussed.

## 2. Effects of TQ-Induced I/R Injury on Localized Skeletal Muscles

TQ application during TKA has been shown to produce I/R injury in human skeletal muscle by triggering cascades of cellular events resulting in a reduction in protein synthesis [9], an increase in protein degradation [10, 11], and an upregulation of the genes in cell stress pathways [12]. Alterations in the protein metabolism as a result of I/R injury lead to the mobilization of free amino acids [11] which subsequently contribute to quadriceps muscle atrophy [3]. Cap-dependent translation initiation and elongation in the protein synthesis pathway were inhibited during ischemia and in early reperfusion phases causing downregulation of protein synthesis and a 12% loss of mid-thigh quadriceps volume as measured by magnetic resonance imaging (MRI) at two weeks after surgery [9]. The ubiquitin (Ub) proteasome system, the main pathway of skeletal muscle proteolysis, was upregulated at 60 minutes of ischemia suggesting an increase in muscle protein breakdown [10]. Regarding the analysis of gene expression profiles following TKA, 72 genes in skeletal muscle cells were significantly upregulated at two hours after TQ release [12]. The genes related to the cell stress pathways were altered and potentially induced apoptosis, cell cycle regulation, and complement activation.

Other mechanisms of skeletal muscle I/R injury have been investigated [13, 14]. Many studies [15–17] have reported that endothelial dysfunction resulting from an imbalance of vasoactive substances, including endothelin 1 (ET-1), as well as neuronal and endothelial nitric oxide synthases (nNOS and eNOS) plays a role in the pathophysiology of several ischemic conditions. Concordantly, ET-1, nNOS, and eNOS are involved in skeletal muscle I/R. The rise in ET-1 tissue protein levels occurred during the periods of I/R and was attributed to an increase in the release of stored peptides or the conversion of precursor peptides to ET-1 [13]. Furthermore, the upregulation of NOSs occurred in postischemic skeletal muscle. The increased protein expression of nNOS was controlled at the mRNA level, whereas the upregulation of the eNOS protein was regulated by posttranscriptional processes [14]. All these findings suggest that agents modulating the ET-1 and NO pathways such as an ET antagonist may have therapeutic benefits in this condition.

The cellular bioenergetics and mitochondria are preserved during skeletal muscle I/R in the TKA setting [11, 18, 19]. For example, (1) adenosine triphosphate (ATP) concentrations and mitochondrial enzymes were maintained during 60–90 minutes of ischemic time and at 24 hours after reperfusion [11, 18], despite significant metabolic changes which suggested ischemic injury to the skeletal muscle cell at approximately 75 minutes of ischemia [20], and (2) a previous human study [19] showed mitochondria to have normal appearance when viewed under an electron microscope after 15 minutes of ischemia. However, in cases of myocardial I/R condition, mitochondrial respiratory chain activity was reduced after 30 minutes of ischemia and restored upon reperfusion as a biphasic process [21, 22]. All those findings suggest that alterations in mitochondrial function induced by I/R injury are tissue-specific and the severity of the cellular damage depends on the duration of ischemia. However, the actual period of total ischemia which results in mitochondrial damage and the reversal time of mitochondrial dysfunction have not yet been validated. Therefore, the ischemic time inducing skeletal muscle mitochondrial impairment should be further defined and therapeutic strategies to address the prevention and modulation of mitochondrial injury should be studied. A comprehensive summary of those findings is shown in Table 1.

## 3. Effects of TQ-Induced I/R Injury on Local and Systemic Circulation

TQ use can lead to a production of oxygen free radicals and stimulation of inflammatory processes in the ischemic skeletal muscle cells and endothelium. Upon TQ release, activated endothelial cells generate more oxygen free radicals and release inflammatory mediators [23]. The elevated oxidative stress levels and the inflammatory reaction in both the local and systemic circulation after TQ deflation were demonstrated in the TKA-related I/R models [24–26]. Interestingly, the changes were observed earlier and more intensely in the blood from the reperfused area than from the systemic circulation. The rise in systemic prooxidant and hypoxanthine levels as well as in xanthine oxidase activity is probably explained by a dispersal of these molecules from the injured area into the systemic circulation because hypoxanthine accumulates in hypoxic conditions [25]. On the other hand, the circulatory increase in pro- and anti-inflammatory cytokines can be explained by systemically induced stress responses secondary to tissue trauma. The number and type of lymphocytes can be used to monitor the systemic effect of the stress response, and the application of a TQ has been shown to induce genotoxic and cytotoxic effects on peripheral leukocytes during the reperfusion period with possible irreversible damage [27]. Despite these acknowledged deleterious effects of use of a TQ, the surgical trauma per se generates surgical stress which is characterized by neuroendocrine, immunological, and hematological changes. When compared to the procedure without TQ application, the increase in plasma interleukin-6 (IL-6), C-reactive protein (CRP), creatine phosphokinase (CPK) and white blood cell counts at 24 hours and seven days after surgery were not different and improvement of knee function at one year after operation was comparable [28]. It is possible that these long-term systemic responses originated from the surgical injury. Further studies focusing on differentiation between responses from surgical stress and those from I/R injury in this setting should be investigated. A comprehensive summary of those findings is shown in Table 2.

## 4. Effects of TQ-Induced I/R Injury on Remote Organs

The I/R of the lower extremity affects not only the local structures but also distant organs. The remote response to I/R is associated with microvascular dysfunction [23]. Activated endothelial cells produce excessive ROS at the initiation of reperfusion and lead to an imbalance between superoxide and nitric oxide in all segments of the microcirculation, which subsequently induce a systemic inflammatory response and cause multiple organ damage. A previous study [29] reported hepatic and renal dysfunction as well as pulmonary damage in animals subjected to three hours of bilateral hind limb ischemia, followed by three hours of reperfusion. In relation to TQ-induced I/R in the clinical setting, remote kidney damage was suggested by the elevation of two sensitive indicators of proximal tubular function [30]. However, no significant myocardial, cerebral, or lung injury was demonstrated after unilateral TKA surgery [31–33]. It is likely that the severity of distant organ injury is related to the degree of local tissue injury and systemic inflammatory activation. This supposition is supported by higher postoperative complications affecting multiple organ systems among bilateral TKA patients compared to those undergoing a unilateral TKA [34]. A comprehensive summary of these findings is shown in Table 3.

## 5. Effects of Ischemic Conditioning on TQ-Induced I/R Injury in TKA

Ischemic preconditioning (IPC) is an exposure of tissues to one or more brief periods of I/R which generates small amounts of free radicals resulting in an adaptive response to subsequent prolonged ischemic stress and reperfusion injury [35]. The IPC results in protection, consisting of two phases, an early phase and a late phase [23, 36]. The early phase affects ion channel permeability, posttranslational modification of proteins, and release of autocoids such as adenosine, bradykinin, and nitric oxide. The later phase is dependent on the gene expression and de novo protein synthesis involved in endothelial function, an inflammatory response, and hemostasis. During the conditioning, the released autocoids bind to G-protein-coupled receptors (GPCRs) which subsequently activate growth factor receptors (GFRs) and in addition stimulate intracellular kinase pathways. These processes result in an increase in antiapoptotic proteins, inhibition of proapoptotic proteins, translocation of transcription factors, opening of ATP-sensitive potassium channels (K_ATP_), and inhibition of the mitochondrial permeability transition pores (mPTPs). The IPC of the lower extremity in unilateral TKA patients showed protective genomic responses, which resulted in an upregulated expression of immediate early response genes, oxidative stress defense genes and prosurvival genes, and regulation of neuron apoptosis [37, 38]. However, the systemic inflammatory signals were not suppressed by IPC performed with one to three cycles of five-minute ischemia and five-minute reperfusion [33, 37, 39]. A comprehensive summary of these findings is shown in Table 4.

Remote ischemic preconditioning (rIPC) is the conditioning applied to distant tissues or organs in order to render tissues with a subsequent sustained ischemic episode resistant to I/R injury. The potential mechanisms of rIPC consist of two components which are humoral and neural [40, 41]. The two hypotheses involve production of endogenous substrates, such as adenosine, bradykinin, and calcitonin gene-related peptides (CGRP) in the remote ischemic tissues. These endogenous mediators enter the bloodstream and initiate protective effects *via* their respective receptors in other tissues. In a different way, these substrates stimulate afferent nerve fibers and transmit protection to distant organs through efferent nerve fibers. Thus, an intact neural pathway is required for the complete signaling of remote preconditioning. The skeletal muscle ischemia resulting from use of a TQ on a nonoperated thigh has been investigated in the TKA setting. This rIPC with three cycles of five-minute ischemia improved regional cerebral and pulmonary oxygenation during the early reperfusion period in the patients undergoing unilateral TKA under general anesthesia [32]. However, in cases of bilateral TKA, application of a thigh TQ in the first-operated knee may prevent I/R injury from occurring during the subsequent ischemic surgical procedure on the other knee [31, 42, 43]. Nonetheless, the conditioning stimulus of rIPC in these previous studies was unclear. The ischemic times of the preconditioning, of approximately 60–90 minutes, were longer than a typical ischemic stimulus of IPC. It is uncertain whether a longer conditioning time is more effective than a conventional time [44]. It is noteworthy that the anesthesia technique should be focused because spinal anesthesia can block neural impulses at spinal nerve roots and may interfere with the neural pathway of rIPC. A comprehensive summary of these findings is shown in Table 4.

## 6. Effects of Anesthetic Agents on TQ-Induced I/R Injury in TKA

Anesthetic intervention to reduce TQ-related I/R injury in cases of orthopedic surgery has been systematically reviewed [8]. Anesthetic agents with proven antioxidative effects include propofol, dexmedetomidine, and ketamine. Intravenous propofol (2,6-diisopropylphenol) is a common choice as an anesthetic agent for sedation and maintenance of anesthesia. Its antioxidative properties arise from its chemical structure which is similar to the endogenous antioxidant *α*-tocopherol and phenol-based free radical scavengers [45]. The cardioprotective effect of propofol in cases of cardiac I/R is dose-dependent and mediated by the activation of mitochondrial respiratory chain complexes [46, 47]. However, in skeletal muscle I/R, the small or sedation dose of propofol (2 mg/kg/h) infused throughout the operation demonstrated antioxidant and anti-inflammatory properties [48, 49]. Sevoflurane and other halogenated volatile anesthetics have shown protective effects on the myocardium after cardiac I/R [50]. However, the antioxidative effect of sevoflurane and halothane were less than intravenous propofol in this skeletal muscle I/R setting [51, 52]. Therefore, a reasoned anesthetic technique for TKA with TQ is a combined spinal anesthesia with small-dose propofol infusion [48, 49, 51–53]. A role of peripheral nerve blockade for post-TKA pain control has received increasing attention, but its effects on oxidative stress and inflammatory responses have not been investigated. A comprehensive summary of these findings is shown in Table 5.

## 7. Effects of Pharmacological Intervention on TQ-Induced I/R Injury in TKA

ROS from the TQ-related I/R can be modulated by antioxidants. The antioxidants may reduce the cellular level of oxygen free radicals either by inhibiting ROS production, enhancing antioxidant enzymes, or reacting with the free radical intermediates in chain reactions [54]. Besides the antioxidants, interventions preventing mitochondrial dysfunction and local and systemic inflammation processes possibly play an important role in skeletal muscle I/R protection.

Previous studies [11, 30, 31, 53, 55] concerning the preventive effects of vitamin C, mannitol, *N*-acetylcysteine (NAC), inhaled nitric oxide (iNO) and a low concentration of oxygen on I/R injury following TKA have been investigated. Vitamin E and vitamin C are natural nonenzymatic antioxidants that effectively scavenge lipid peroxyl radicals and terminate the lipid peroxidase chain reaction [56]. Administrated intravenously for ten minutes before TQ deflation and 20 minutes after reperfusion, high-dose vitamin C significantly reduced serum malondialdehyde (MDA) levels, a toxic metabolite of lipid peroxidation. Furthermore, vitamin C showed protective effects on the myocardium by significantly reducing troponin I levels at eight hours after the operation compared to the level observed in the controls [31]. Mannitol, a scavenger of hydroxyl free radicals, did not decrease the effects of reperfusion injury on skeletal muscle [11] although a dose-dependent attenuation of oxidative stress induced lung injury following liver I/R has been reported [57]. The exogenous administration of NO lessened the reperfusion inflammatory response in knee surgery patients having general anesthesia [58]. However, with the spinal anesthesia technique, neither local nor systemic signs of endothelial cell activation or inflammatory response were detected at two hours after TQ release. Therefore, the presence of intraoperative iNO did not have a positive effect in this setting [55]. A lower oxygen tension during spinal anesthesia may be an explanation because the formation of isofurans, a free radical mediated peroxidation of arachidonic acid, increased concomitantly with elevated O_2_ concentrations occurring during general anesthesia [53]. Regarding NAC, it is a direct precursor to glutathione (GSH) which directly scavenges ROS and indirectly supports GSH peroxidase [59]. The beneficial effect of NAC on TQ-related I/R injury has been reported [60, 61]. However, a high dose of NAC significantly increased urine markers indicating renal tubular damage [30]. Therefore, techniques of administration including optimal dose, route, and timing of pharmacological interventions should be carefully validated in the skeletal muscle I/R model. A comprehensive summary of these findings is shown in Table 6.

## 8. Conclusions

Use of a TQ during TKA resulted in skeletal muscle I/R injury to localized skeletal muscle, systemic circulation, and distant organs. In the skeletal muscle, changes in protein metabolism suggest inhibition of protein synthesis and enhancement of protein breakdown. During I/R, genes related to the cell stress pathways are upregulated in skeletal muscle cells without evidence of mitochondrial dysfunction. In terms of circulation, oxidative injuries and inflammatory responses are more intense in the reperfused area than in the systemic circulation. As regards remote organs, no significant myocardial, cerebral, or lung injuries were reported but the renal proximal tubular function was impaired.

Several studies investigated the protective effects of IPC, anesthetic agents, and other pharmacological interventions. Sedative doses of propofol have antioxidative and anti-inflammatory properties. However, biochemical outcomes of the use of IPC and other medication to prevent I/R damage were diversified depending on the technique of administration. The optimal technique of therapeutic interventions and the biochemical results thereof should be further verified and correlated to clinical outcomes after TKA.

**Figure 1:**
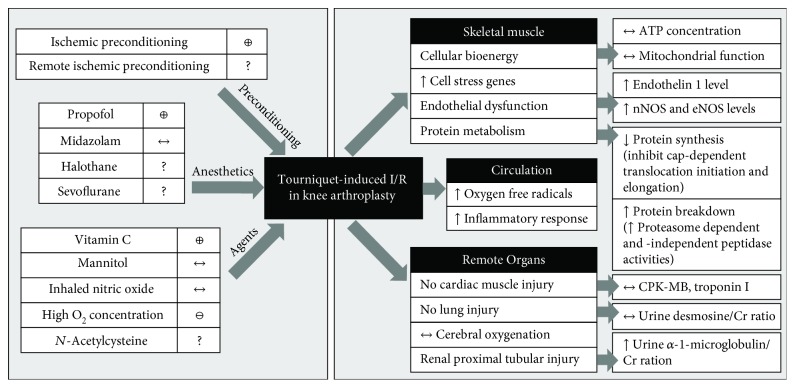
Effects of tourniquet- (TQ-) induced I/R injury on localized skeletal muscle, circulation, and remote organs and the effects of therapeutic interventions on the skeletal muscle I/R in cases of surgery for knee arthroplasty (TKA). The skeletal muscle I/R condition results in (1) preserved cellular bioenergy and mitochondrial function, (2) upregulation of genes related to cell stress pathways, (3) endothelial dysfunction as indicated by an increase in endothelin 1 and NOS levels, (4) alteration in protein metabolism, (4) increased oxidative stress and inflammatory responses, and (5) injury to distant organs including the kidney. Ischemic preconditioning (IPC), propofol, and vitamin C demonstrated positive or protective effects in the cases of I/R injury in this setting, while elevated O_2_ tension aggravated the injury and *N*-acetylcysteine may have dose-dependent responses. Other interventions including remote ischemic preconditioning (rIPC), volatile anesthetic agents, mannitol, and nitric oxide possibly produce positive outcomes, and additional studies in this I/R condition should be investigated. ⊕: positive effect; ⊖: negative effect; ?: inadequate evidence; ↑: increase; ↓: decrease; ↔: no change; ATP: adenosine triphosphate; CPK-MB: creatinine phosphokinase-MB; Cr: creatinine; eNOS: endothelial nitric oxide synthase; nNOS: neuronal nitric oxide synthase; O_2_: oxygen.

The effects of TQ-induced I/R injury on localized skeletal muscle, circulation, and remote organs and the effects of therapeutic interventions on the skeletal muscle I/R in TKA are summarized in Figure 1.

## Figures and Tables

**Table 1:** Effects of TQ-induced I/R injury on localized skeletal muscles.

Study model/specimen/sample size/age	TQ pressure/ischemia time	Major findings (compared to baseline level)			Interpretation	References
		Clinical outcome	Mechanism			
			Ischemia phase	Reperfusion phase		
Cross-sectional study/vastus lateralis muscle biopsy/*n* = 13/62–76 yr	≥300 mmHg, 32–52 min	↓ 12% of quadriceps muscle volume at 2 weeks after surgery	↔ eIF4G gene expression ↓ phosphorylation of Akt at Ser^473^ and 4E-BP1 ↑ phosphorylation of eEF2	↑ eIF4G gene expression ↓ phosphorylation of Akt at Ser^473^ and 4E-BP1 ↑ phosphorylation of eEF2	Cap-dependent translation initiation and elongation may be inhibited during skeletal muscle I/R	[9]
Randomized controlled trial/vastus medialis muscle biopsy/*n* = 34/55–85 yr	380 mmHg, 60 min	N/A	↔ free, conjugated Ub ↔ total Ub-protein ligase activity ↑ proteasome-dependent and -independent peptidase activities	N/A	Upregulated proteasome-dependent and -independent peptidase activities suggested an increase in protein degradation at 60-minute ischemia time	[10]
Randomized controlled trial/vastus lateralis muscle biopsy/*n* = 15/63–76 yr	Double SBP mmHg, 56–92 min	N/A	↑ all muscle-free amino acids, except glutamate ↔ mitochondrial enzymes	↓ all muscle-free amino acids ↔ mitochondrial enzymes	Degradation of free amino acids was more than synthesis during skeletal muscle ischemia No mitochondrial dysfunction occurred at maximum ischemia and at 24 hours after reperfusion	[11]
Cross-sectional study/vastus lateralis muscle biopsy/*n* = 13/60–78 yr	≥300 mmHg, 33–50 min	N/A	N/A	Upregulation of 72 genes including JAK-STAT, p53, JNK, NF*κ*B, Akt, and MAPK	Genes related to cell stress pathways involved in reperfusion injury	[12]
Cross-sectional study/quadriceps muscle biopsy, venous blood/*n* = 13/64–89 yr	300 mmHg	N/A	↑ positive ET-1-immunostaining cells ↔ ET-1 mRNA expression ↔ plasma ET-1	↑ positive ET-1-immunostaining cells ↔ ET-1 mRNA expression ↔ plasma ET-1	ET-1 is involved in skeletal muscle I/R	[13]
Cross-sectional study/quadriceps muscle biopsy/*n* = 12/64–89 yr	300 mmHg	N/A	↔ nNOS, iNOS, and eNOS immunostaining in muscle fibers ↑ nNOS mRNA expression ↔ nNOS and eNOS protein expression ↔ NOS activity	↑ nNOS and eNOS immunostaining in muscle fibers ↑ nNOS mRNA expression ↑ nNOS and eNOS protein expression ↔ NOS activity	nNOS and eNOS were upregulated in postischemic muscle, but their activities were not altered	[14]
Randomized controlled trial/interstitial space fluid at gastrocnemius muscle/*n* = 31/68 ± 8 yr	250 mmHg, 74 ± 4 min	N/A	↓ glucose ↓ pyruvate ↑ lactate ↑ L/P ratio ↑ glycerol	↓ glucose ↑ pyruvate ↑ lactate ↔ L/P ratio ↑ glycerol	Changes in the level of metabolic markers in the extracellular space suggested ischemic injury and persisted for up to 180 minutes after reperfusion	[20]
Randomized controlled trial/vastus medialis muscle biopsy/*n* = 10/74 ± 3 yr	380 mmHg, 60 min	N/A	↔ mitochondrial enzymes	N/A	No effects on amount and function of mitochondria at 60-minute ischemia time	[18]

4E-BP1: eukaryotic initiation factor 4E-binding protein; eEF2: eukaryotic elongation factor 2; eIF4G: eukaryotic translation initiation factor 4 gamma; ET-1: endothelin 1; I/R: ischemia and reperfusion; JAK-STAT: Janus kinase/signal transducer and activator of transcription; JNK: c-Jun N-terminal kinase; L/P: lactate/pyruvate; MAPK: mitogen-activated protein kinase; mRNA: messenger ribonucleic acid; N/A: not available; NF*κ*B: nuclear factor kappa-light-chain-enhancer of activated B cells; NOS: nitric oxide synthase; SBP: systolic blood pressure; TQ: tourniquet; Ub: ubiquitin.

**Table 2:** Effects of TQ-induced I/R injury on local and systemic circulation.

Study model/specimen/sample size/age	TQ pressure/ischemia time	Main findings (compared to baseline level)			Interpretation	References
		Clinical outcome	Mechanism			
			Local circulation	Systemic circulation		
Randomized controlled trial/antecubital venous blood/*n* = 15/blood from surgical drain/*n* = 17/approx. 70 ± 7 yr	250 mmHg, approx. 90 ± 15 min	N/A	Surgical drainage tube ↓↓ GSH ↑↑ GSSG ↑↑ MDA	Antecubital vein ↓ GSH ↑ GSSG ↑ MDA	Changes in glutathione oxidation and lipid peroxidation happened earlier and more intensely in the blood from the reperfused area than from the systemic circulation	[24]
Cross-sectional study/antecubital and femoral venous blood/*n* = 10/69 ± 2 yr	Double SBP mmHg, 85 ± 8 min	N/A	Femoral vein of operated leg ↑↑ hypoxanthine, XO activity, and xanthine ↑ GSSG/GSH	Antecubital vein ↑ hypoxanthine, XO activity, and xanthine ↔ GSSG/GSH	Higher increase in prooxidants and oxidative stress in the blood from the reperfused area compared to the systemic circulation	[25]
Cross-sectional study/great saphenous venous blood of both legs, blood from surgical drain/*n* = 9/57–71 yr	250–300 mmHg, 78–125 min	N/A	Surgical drainage tube ↑↑ IL-6 ↑ IL-10	Great saphenous vein of nonoperated leg ↑ IL-6 ↔ IL-10	Higher increase in pro- and anti-inflammatory cytokines in the blood from the reperfused area compared to the systemic circulation	[26]
Cross-sectional study/peripheral blood lymphocytes/*n* = 11/60–75 yr	100–120 min	N/A	N/A	↑ genotoxicity index ↑ cytotoxicity index ↔ cytostaticity index	Genotoxic and cytotoxic effects on peripheral lymphocytes were most pronounced at onset of reperfusion and remained so 1 hour afterward	[27]
Case-control study/venous blood/*n* = 20/74 ± 7 yr	250–300 mmHg, 80 ± 20 min	↑ knee function scores No surgical site infection at 1 yr	N/A	↑ IL-6, CPR, CPK, and white cell count	Oxidative stress after TKA surgery primarily originated from surgical stress only	[28]

CPK: creatine phosphokinase; CRP: c-reactive protein; GSH: reduced glutathione; GSSG: oxidized glutathione; IL: interleukin; I/R: ischemia and reperfusion; MDA: malondialdehyde; N/A: not available; SBP: systolic blood pressure; TKA: total knee arthroplasty; TQ: tourniquet; XO: xanthine oxidase.

**Table 3:** Effects of TQ-induced I/R injury on remote organs.

Sample size/age	TQ pressure/ischemia time	Main findings (compared to baseline level)		Interpretation	References
		Outcomes on remote organ	Systemic effects		
*n* = 16/70 ± 4 yr	91 ± 11 min, N/A	Heart: ↔ CPK-MB ↔ Troponin I	↑ serum MDA	No cardiac muscle injury after TKA with TQ	[31]
*n* = 36/71 ± 7 yr	N/A	Brain: ↔ rScO_2_ No POCD at 1 week Lungs: ↔ PF ratio Kidney: ↔ serum Cr	↑ plasma lactate ↑ serum CPK ↔ serum LDH, AST ↔ serum IL-6, TNF-*α*, IL-10 ↓ serum TNF-*β*	No adverse effects on regional cerebral oxygenation, pulmonary oxygenation, and renal function after TKA with TQ	[32]
*n* = 17/67 ± 10 yr	250 mmHg, 52 ± 11 min	Lungs: ↔ urine desmosine/Cr ratio	↑ serum IL-6, TNF-*α*, CRP, and WBC count	No lung injury occurred as indicated by the unaltered marker of elastin breakdown after TKA with TQ	[33]
*n* = 15/64–73 yr	300–350 mmHg, 83–121 min	Kidney: ↑ urine *α*-1-microglobulin/Cr ratio ↑ urine GST-*α*/Cr ratio ↔ urine NAG/Cr ratio ↓ serum cystatin C ↓ serum Cr and urea	↑ plasma lactate ↑ serum myoglobin ↑ serum lactoferrin	Possible proximal tubular injury after TKA with TQ	[30]

AST: aspartate aminotransferase; CPK: creatinine phosphokinase; CRP: c-reactive protein; Cr: creatinine; GST-*α*: glutathione-S-transferase-*α*; IL: interleukin; LDH: lactate dehydrogenase; MDA: malondialdehyde; N/A: not available; NAG: *N*-acetyl-*β*-D-glucosaminidase; PF ratio: ratio of arterial oxygen partial pressure to fractional inspired oxygen; POCD: postoperative cognitive dysfunction; rScO_2_: regional cerebral oxygen saturation; TKA: total knee arthroplasty; TNF: tumor necrosis factor; TQ: tourniquet; WBC: white blood cell.

**Table 4:** Effects of ischemic preconditioning (IPC) and remote IPC (rIPC) on TQ-induced I/R injury in TKA.

Study model/specimen/TQ pressure	IPC protocol/TQ ischemia time/sample size/age		Main findings (compared to control)		Interpretation	References
	Intervention	Control	Clinical outcome	Mechanism		
RCT/antecubital venous blood, quadriceps muscle biopsy, SBP + 100 mmHg	3 cycles of 5 min ischemia and 5 min reperfusion at operated thigh, 68–87 min, *n* = 10	no IPC, 68–87 min, *n* = 10	N/A	↑ gene expression (i) immediate early response genes (ii) oxidative stress defense genes (iii) mitochondrial genes (iv) prosurvival genes ↓ gene expression (i) proapoptotic genes ↔ serum IL-6, CRP, ESR, and WBC count	IPC induced a protective genomic response IPC did not prevent systemic inflammatory response	[37]
Case-control study/quadriceps muscle biopsy	N/A, *n* = 4	No IPC, *n* = 4	N/A	Altered expression of genes involved in neurological system process and regulation of neuron apoptosis	IPC induced a protective genomic response	[38]
RCT/venous blood, urine/250 mmHg	1 cycle of 5 min ischemia and 5 min reperfusion at operated thigh, 58 ± 11 min, *n* = 17, 67 ± 11 yr	no IPC, 52 ± 11 min, *n* = 17, 67 ± 10 yr	↓ median pain scores within 48 h after surgery ↔ postoperative analgesic consumption ↓ length of hospital stay	↔ serum IL-6, TNF-*α*, CRP, and WBC count ↔ urine desmosine/Cr ratio	IPC did not prevent systemic inflammatory response or the level of lung injury IPC may improve postoperative pain control	[33]
RCT/venous blood, blood from surgical drain/250 mmHg	1 cycle of 5 min ischemia and 5 min reperfusion at operated thigh, 48 min (IQR 13), *n* = 30, 67 yr (IQR 10.8)	no IPC, 54 min (IQR 18), *n* = 30, 72.5 yr (IQR 13)	↓ pain scores within 48 h after surgery ↔ postoperative analgesic consumption ↔ physical therapy parameters ↔ length of hospital stay	↔ intraarticular IL-6, TNF-*α* ↔ systemic prothrombotic levels	IPC may improve postoperative pain control Hypercoagulative state occurred after TKA surgery using TQ application	[39]
RCT/arterial blood, venous blood/double SBP mmHg	3 cycles of 5 min ischemia at nonoperated thigh, *n* = 36, 69 ± 7 yr	No IPC, *n* = 36, 71 ± 7 yr	Brain: ↑ rScO_2_ ↔ POCD at 1 week Lungs: ↑ PF ratio	↓ serum LDH ↔ serum CPK and AST ↔ serum IL-6, TNF-*α*, IL-10, and TNF-*β*	Remote IPC improved regional cerebral and pulmonary oxygenation possibly via a decrease in tissue damage	[32]
Cross-sectional study/venous blood/double SBP mmHg	Approx. 60 min ischemia at previously operated thigh, 62 ± 19 min, *n* = 12, 67 ± 5 yr	First-operated knee, 63 ± 14 min, *n* = 12, 67 ± 5 yr	N/A	Tend to ↓ whole blood ROS production ↔ plasma PCOOH	Remote IPC may occur during bilateral TKA with sequential application of TQ	[42]
Cross-sectional study/venous blood	Approx. 90 min ischemia at previously operated thigh, 89 ± 9 min, *n* = 16, 70 ± 4 yr	First-operated knee, 91 ± 11 min, *n* = 16, 70 ± 4 yr	N/A	Tend to ↓ serum MDA	Remote IPC may occur during bilateral TKA with sequential application of TQ	[31]
Cross-sectional study/venous blood (dorsum of each foot)/double SBP mmHg	Approx. 60 min ischemia at previously operated thigh with 20 min reperfusion, 62 ± 19 min, *n* = 30, 64 ± 5 yr	First-operated knee (right), 61 ± 5 min, *n* = 30, 64 ± 5 yr	Muscle: ↔ WOMAC scores (assessment of joint pain, stiffness, and function) at 1 month	↔ serum MDA ↔ serum LDH	Sequential ischemic surgical procedure did not reduce oxidative injury after reperfusion	[43]

AST: aspartate aminotransferase; CPK: creatinine phosphokinase; CRP: c-reactive protein; Cr: creatinine; IL: interleukin; ESR: erythrocyte sedimentation rate; IPC: ischemic preconditioning; I/R: ischemia and reperfusion; IQR: interquantile range; LDH: lactate dehydrogenase; MDA: malondialdehyde; N/A: not available; PCOOH: phosphatidylcholine hydroperoxide; PF ratio: ratio of arterial oxygen partial pressure to fractional inspired oxygen; POCD: postoperative cognitive dysfunction; RCT: randomized controlled trial; ROS: reactive oxygen species; rScO_2_: regional cerebral oxygen saturation; SBP: systolic blood pressure; TKA: total knee arthroplasty; TNF: tumor necrosis factor; TQ: tourniquet; WBC: white blood cell; WOMAC: Western Ontario and McMaster University Osteoarthritis Index.

**Table 5:** Effects of anesthetic agents on TQ-induced I/R injury in TKA.

Study model/specimen/TQ pressure	Anesthetic agent/dose/TQ ischemia time/sample size/age		Main findings (compared between groups)		Interpretation	References
	Intervention	Control	Clinical outcome	Mechanism		
RCT/venous blood/350 mmHg	Propofol, 2 mg/kg/h, 90 ± 7 min, *n* = 18, 66 ± 7 yr	Normal saline, 0.2 ml/kg/h, 93 ± 10 min, *n* = 17, 69 ± 10 yr	Sedation effect Propofol > control	Plasma SOD, TCA Propofol > control Serum MDA, hsCRP, and blood neutrophil count Propofol < control	Sedation dose of propofol has antioxidative and anti-inflammatory properties	[48]
RCT/arterial blood/double SBP mmHg	Propofol, 0.2 mg/kg then 2 mg/kg/h, 72 ± 18 min, *n* = 11, 67 ± 5 yr	Midazolam, 5 mg, 69 ± 14 min, *n* = 11, 63 ± 7 yr	N/A	Whole blood ROS production Propofol < midazolam	Sedation dose of propofol attenuates ROS production compared to midazolam	[49]
RCT/venous blood	Propofol, 2–2.5 mg/kg then 6–10 mg/kg/h, 79 ± 13 min, *n* = 10, 70 ± 6 yr	Sevoflurane, 1.5–2%, 83 ± 15 min, *n* = 10, 69 ± 5 yr	N/A	Serum MDA Propofol < sevoflurane	Propofol reduces oxidative injury in the TQ-induced I/R model	[51]
RCT/arterial blood, venous blood/350–400 mmHg	Propofol, 2 mg/kg then 4–8 mg/kg/h, 114 ± 19 min, *n* = 15, 69 ± 6 yr	Halothane, 0.7–1%, 116 ± 25 min, *n* = 15, 66 ± 5 yr	MAP, pH, PaO_2_, PaCO_2_ Propofol ↔ halothane	Serum MDA Propofol < halothane	Propofol reduces oxidative injury in the TQ-induced I/R model	[52]

hsCRP: high-sensitivity C-reactive protein; I/R: ischemia and reperfusion; MAP: mean arterial pressure; MDA: malondialdehyde; N/A: not available; PaCO_2_: arterial carbon dioxide partial pressure; PaO_2_: arterial oxygen tension; RCT: randomized controlled trial; ROS: reactive oxygen species; SBP: systolic blood pressure; SOD: superoxide dismutase; TAC: total antioxidative capacity; TQ: tourniquet.

**Table 6:** Effects of pharmacological intervention on TQ-induced I/R injury in TKA.

Study model/specimen/TQ pressure	Drug/dose/TQ ischemia time/sample size/age		Main findings (compared between groups)	Interpretation	References
	Intervention	Control			
RCT/venous blood	Vitamin C, 0.03 g/kg during ischemia then 0.01 g/kg after reperfusion, 91 ± 11 min, *n* = 16, 71 ± 4 yr	Normal saline, 91 ± 14 min, *n* = 16, 70 ± 4 yr	Serum MDA Serum troponin I Vitamin C group < control	High-dose vitamin C prevents oxygen free radical production and may have myocardial protection properties	[31]
RCT/vastus lateralis muscle biopsy/double SBP mmHg	Mannitol, 930 mosmol/kg, 12.5 mL/kg/day, 75–93 min, *n* = 8, 64–74 yr	5% glucose, 18.75 mL/kg/day, 50–88 min, *n* = 7, 62–79 yr	Muscle GSH, tGSH Muscle amino acid Mannitol group ↔ control	No positive effects of mannitol in this TQ-induced I/R model	[11]
RCT/venous blood, quadriceps femoris muscle biopsy/300 mmHg	iNO, 80 ppm entire operation, 101 ± 20 min, *n* = 15, 63 ± 14 yr Partial iNO, 80 ppm during operation except ischemia period, 103 ± 19 min, *n* = 15, 65 ± 9 yr	Nitrogen, 95 ± 19 min, *n* = 15, 64 ± 9 yr	Plasma ICAM, VCAM Plasma P-selectin, E-selectin CD68^+^ macrophage expression Expression of ICAM, VCAM, P-selectin iNO, partial iNO groups ↔ control	No beneficial effects of iNO in this TQ-induced I/R model	[55]
RCT/antecubital venous blood/200 mmHg	Lower O_2_ tension, FiO_2_ = 0.4, 61–110 min, *n* = 19, 66–77 yr	Higher O_2_ tension, FiO_2_ = 0.5, 86–107 min, *n* = 20, 62–74 yr	Plasma isofurans Lower FiO_2_ < higher FiO_2_	Elevated O_2_ tension during general anesthesia reflects increased oxidative stress	[53]
RCT/venous blood, urine/300–350 mmHg	NAC, 150 mg/kg before ischemia then 6.25 mg/kg/h during ischemia, 77–113 min, *n* = 15, 62–77 yr	5% glucose, 83–121 min, *n* = 15, 64–73 yr	Urine *α*-1-microglobulin/Cr ratio Urine NAG/Cr ratio Urine myoglobin NAC group > control	High-dose NAC may aggravate proximal tubular injury	[30]

Cr: creatinine; FiO_2_: fraction of inspired oxygen; GSH: reduced glutathione; ICAM: intercellular adhesion molecule; iNO: inhaled nitric oxide; I/R: ischemia and reperfusion; MDA: malondialdehyde; NAC: *N*-acetylcysteine; NAG: *N*-acetyl-*β*-D-glucosaminidase; RCT: randomized controlled trial; tGSH: total glutathione; TQ: tourniquet; VCAM: vascular adhesion molecule.
